# Influence of the Undercut Anchor Head Angle on the Propagation of the Failure Zone of the Rock Medium

**DOI:** 10.3390/ma14092371

**Published:** 2021-05-02

**Authors:** Józef Jonak, Robert Karpiński, Andrzej Wójcik

**Affiliations:** Department of Machine Design and Mechatronics, Faculty of Mechanical Engineering, Lublin University of Technology, Nadbystrzycka 36, 20-618 Lublin, Poland; a.wojcik@pollub.pl

**Keywords:** undercut anchors, FEM, breakout failure, cone failure, post-installed anchors

## Abstract

The paper presents the results of a numerical analysis (FEM) describing the effect of the undercutting head angle on the formation of the rock mass failure zone during the initial stages of failure propagation. The research was carried out in the context of developing a technology for rock extraction by controlled pull-out of undercut anchors installed in the rock mass. The focus was on the initial stage of crack propagation and its trajectory for anchors embedded at an assumed constant depth and a value of the friction coefficient of the rock against the anchor head. It is shown that smaller angles favor smaller stripping angles and an increased radial impact of the head on the rock material (in the plane perpendicular to the head axis), while the impact of heads with larger angles is found to favor larger fracture penetration angles and faster penetration towards the free rock surface.

## 1. Introduction

Numerical modeling, combined with experiments, enables detailed understanding of the actual behavior of engineering structures and their optimization [1,2,3,4]. Fracture problems of rock and concrete media (especially steel-reinforced ones) are currently analyzed using FEM algorithms, deterministic and probabilistic methods [5] and ABAQUS programs (e.g., [6]). Steel-reinforced and fiber-reinforced materials have a large share in today’s engineering structures. In numerical modeling of fracture issues, it is then necessary to use a 3D computational model in conjunction with a material model of fracture-plastic (the fracture-plastic material model) [7,8,9]. The strength parameters of these materials relevant to numerical modeling include the compressive strength of concrete cubes, the tensile strength of three-point bending test, and the tensile strength of splitting perpendicular and parallel to the direction of fill. Reinforcement of concrete with fibers increases the tensile strength and ductility of concrete. In the case of rock the cracks have a discrete character (unlike in the case of reinforced concrete, where they are often treated as fuzzy (smeared crack model) [10]. Hence, in the modeling of the rock medium the material is often treated as linear, elastic-brittle.

Recently, the use of DEM (distinct element method), which serves to analyze effectively not only the problems of mechanics of grained media, but also the problems of fracture mechanics of rock media or concrete, is developing more and more rapidly [11,12,13]. 

**Figure 1:**
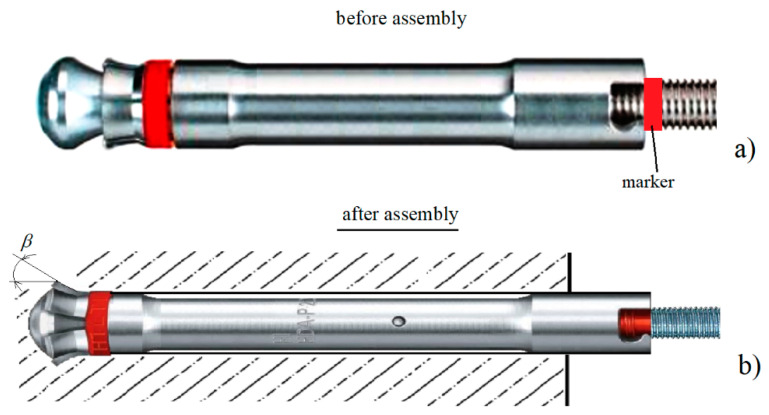
Undercut anchor components (prepared based on Hilti materials): (**a**) anchor in installation configuration with the view of marker location for correct control of anchor head installation, (**b**) anchor head after installation in a hole made in rock, *β*—the angle of taper of working part of undercutting head.

Under typical conditions, undercut anchors (as well as their other designs) are used in the technology of embedment steel structure elements in engineering structures made of concrete (usually of reinforced steel) [14,15,16,17]. The mechanics of concrete deterioration under the action of anchor structures used in fixing techniques is widely described in the literature [14,18,19,20,21]. Among the parameters relevant to the load-bearing capacity of anchors fixed in concrete there are the effective embedment depth, concrete compressive strength, structural dimensions of the anchor head, distance from the edge of the concrete structure, to name a few [22,23,24,25,26]. The shape of the potential failure zone resulting from anchor pull-out is simplified to a cone or pyramid [15,23]. The concrete capacity design (CCD) method considers a rectangular pyramid-shaped failure surface [27,28]. In this method, the tensile concrete capacity of single cast-in and post-installed mechanical anchors is calculated assuming a 35° angle of failure measured from the failure surface to a plane perpendicular to the anchor axis. This gives a horizontal extent of the failure surface approximately three times the effective embedment depth [15,29]. In a different study [30], with the same dimensions of the base and the diameter of the conical neck of the anchor tip, it was found that the smaller the angle of the cone of the anchor head *β* (Figure 1), the higher the load capacity of the anchor (anchor pull-out force).

This paper presents the results of selected analyses carried out with respect to the use of undercut anchors in the tested technology for rock rubble separation [31,32]. This technology is envisaged as an alternative in specific areas of mining activity, e.g., where other mining techniques (the use of explosives or mechanical mining, e.g., shearers) are not possible. Due to the heterogeneity of the internal rock structure, there are certain deviations from the current description of the phenomena of failure of concrete structures, both in terms of the value of the anchor pull-out forces and the extent of the potential failure zone [33].

Under the conditions of field testing, it was considerably difficult to maintain the regime of undercutting anchor installation (Figure 1) imposed by the manufacturer. In order to analyze the influence of this phenomenon on the process of rock medium failure, analyses were performed, the results of which are the subject of the analysis reported herein. For Hilti undercutting anchors, the angle *β* varies between 20° and 30°, depending on the anchor size. The effect of a potential mounting inaccuracy was simulated by varying the head cone angle *β* against a constant value of the cone forming length *L*.

## 2. Analysis

For proper head embedment in a hole drilled in the rock, it is necessary to drill the hole precisely (to maintain the required depth) and to make an undercut with the parameters required by the manufacturer. A suitable marker (Figure 1) is used here to obtain the required position of the resilient sleeve with the undercutting spring elements that are spread out by the special end of the anchor during embedment (undercutting). As a result, the required undercutting angle *β* (Figure 1) and the spreading of undercutting elements of the head in the performed undercutting are obtained. 

**Figure 2:**
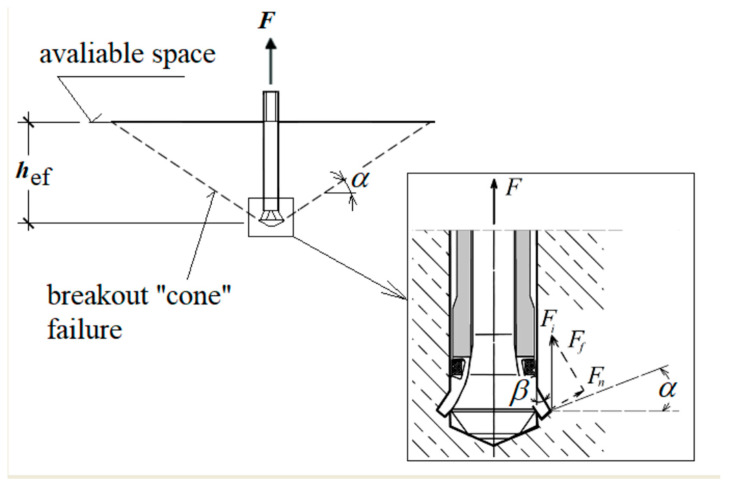
Rock fragmentation with the undercutting head and the distribution of the impact force of a single undercutting element of the anchor head (*F*_i_) on the rock: *F*_n_—normal to the surface of the element; *F*_f_—(friction) tangent to the undercutting element; *α*—the angle of the “cone” of destruction at the initial stage of propagation; *β*—working angle of the undercutting head (cone angle of the undercutting head); *h*_ef_—effective embedment depth; *F*—anchor pull-out force.

Under field test conditions (mining plants), maintaining strict anchor installation regimes is a task of much difficulty. In extreme cases, this can lead to greater or lesser spreading of the undercutting elements of the head. As a result, it is possible for the head to have different undercutting head taper angles *β* (Figure 1 and Figure 2) and different anchor pull-out force (anchor capacity). 

Hirabayashi et al. [30] show that the smaller the cone angle *β*, the higher the anchor capacity (anchor pullout force). According to Furche and Elingehausen [34], smaller anchor head angles result in significantly reduced load capacity. This work shows that for small angles (5° and 20°), the load capacity of the anchors is reduced by as much as 50% compared to the load capacity of anchors with head angles equal to 90°. This topic, therefore, requires more research so as to establish the effect of the head angle *β* on the course and the extent of the potential failure zone during anchor pullout. Due to the design of the current project, this aspect is important for the performance of the planned stripping technology, especially in terms of the interaction of failure zones in multiple anchor systems [35,36]. Hence the interest in the subject matter, which contributed to conducting the analyses, whose results are presented below. 

## 3. Materials and Methods

**Figure 3:**
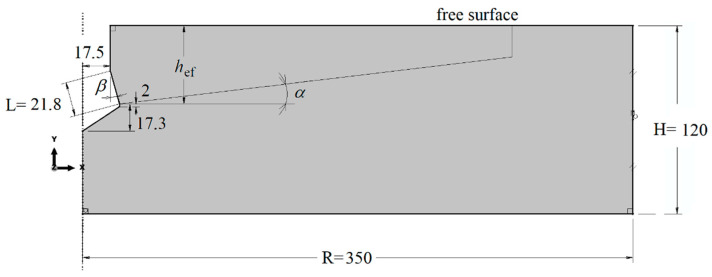
Axisymmetric model of anchor interaction with rock: *β*—angle of anchor head, *α*—potential angle of failure cone, *L*—length of the cone head’s forming line.

The analysis of the effect of the undercutting head cone angle (*β*) on the shape of the failure cone in rock was performed using the FEM ABAQUS system (Abaqus 2019, Dassault Systemes Simulia Corporation, Velizy Villacoublay, France). The initial breakout angle of the fracture/destruction surface was the subject of interest as this is what determines the size of rock prism during the controlled pullout of the undercut anchor. For the purpose of the analysis, an axisymmetric model of the undercut anchor action on the rock medium was adopted, as shown in Figure 3. Model radius R = 350 mm and specimen height H = 120 mm. The embedment depth was set at *h*_ef_ = 50 mm. As can be seen from the dimensions of the model, the ratio *R*/*h*_ef_ was 7 that is substantially larger than it should result from the standard parameters of the breakout prism specified by the CCD method–Cone Capacity Design. Hence, there was no effect of the supports here on the extent of the potential failure cone that is formed in pull-out tests. There was also an effect on how the nodes of the finite element mesh of the model were restrained.

It is further assumed that the length of the conic head of the anchor is constant *L* = *const*, and equal to = 21.8 mm. Under the conditions of field testing the manufacturer-recommended anchor installation process (Figure 1) is rather unattainable. This work reports on the results from the analyzes that aimed to describe the impact of the phenomenon on the rock mass failure process. For Hilti undercutting anchors, the angle *β* varies between 20° and 30°, depending on the anchor size. The effect of potential mounting inaccuracy was simulated, which was achieved by varying the head cone angle *β* against a constant value of the cone-forming length *L* (see Figure 1 and Figure 3). The values of this angle were assumed to be 15°, 20°, 25° and 30°. 

**Figure 4:**
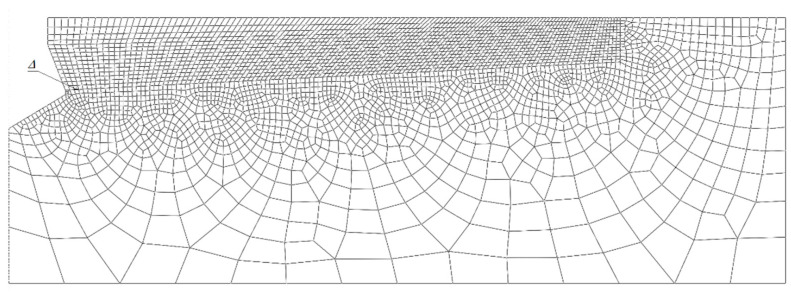
Finite element mesh for an assumed model of anchor action on rock. Δ—the length of the side of the mesh element measured at the side of the potential cone of destruction.

The model takes into account the existence of a potential destruction prism *α* (Figure 2 and Figure 3). Its value was initially estimated in former analyses [31,35,36,37]. For such an adopted model of anchor impact on the rock, a finite element mesh model was obtained as in Figure 4. 

**Figure 5:**
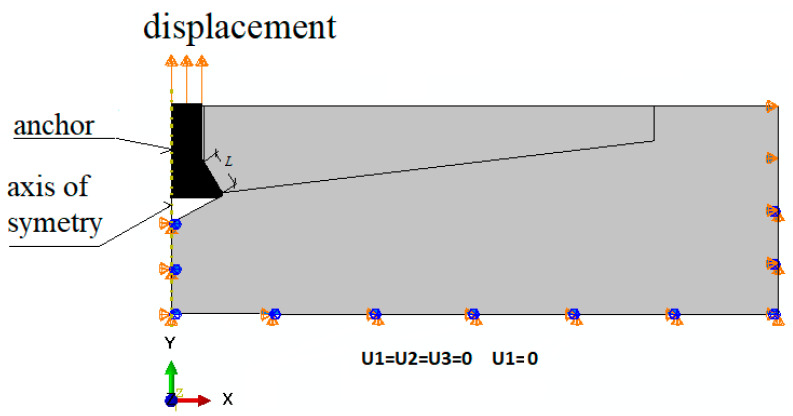
Restraints and Extrusions. *L*—length of the contact zone between anchor head and rock. Discretization method: surface to surface.

The anchor was subject to a controlled displacement (force) along the vertical axis (Figure 5). Between the conical head of the anchor and the rock, a contact was established along the cone’s forming line *L* (ABAQUS–discretization method: surface to surface; finite sliding).

The nodes in the vertical axis of the model under the anchor and at the periphery of the model were deprived of all degrees of freedom (U1 = U2 = U3).

**Figure 6:**
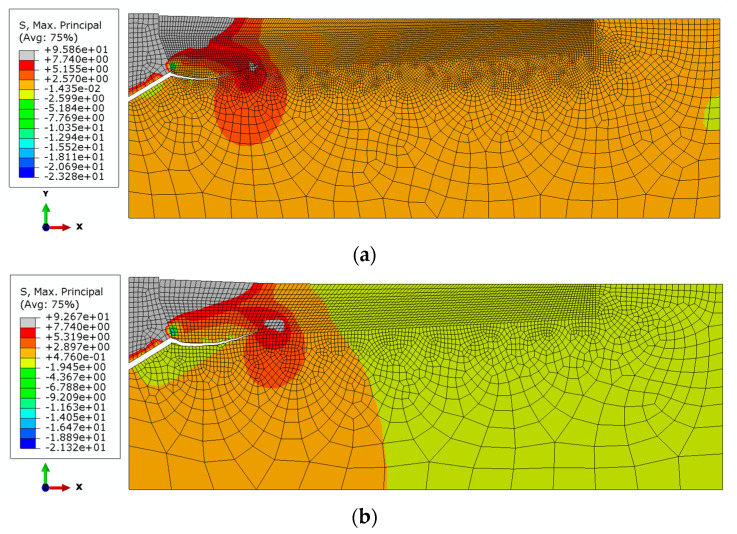

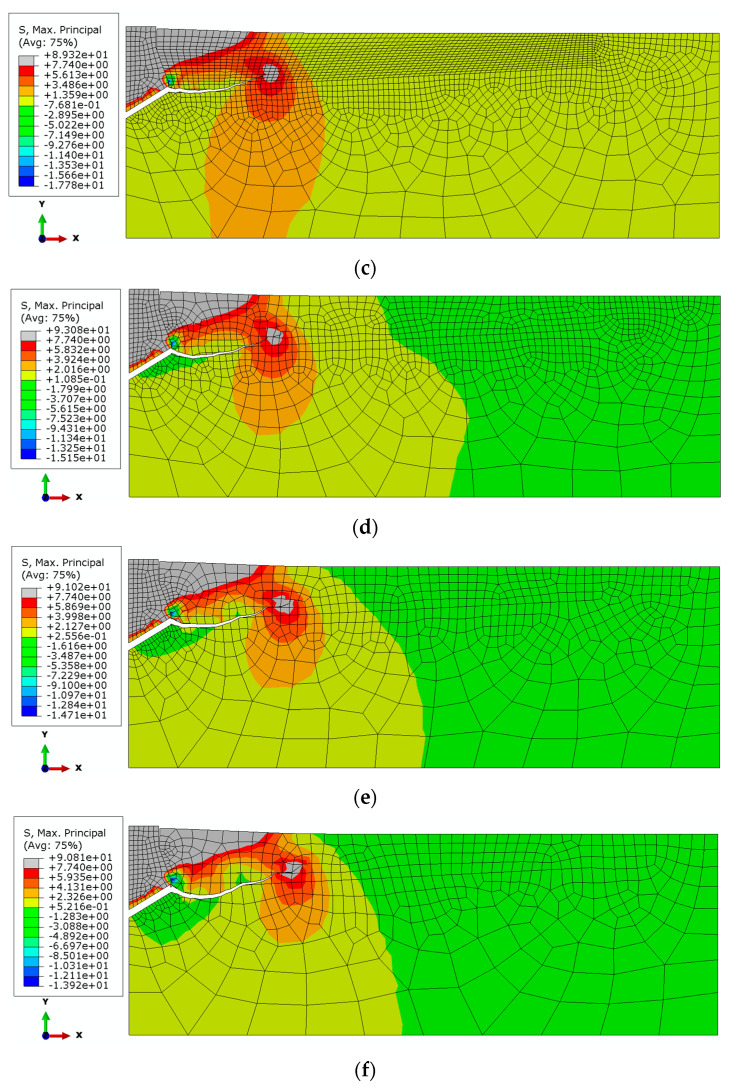
Effect of grid size Δ on the failure trajectory of a rock medium: (**a**) Δ = 1 mm, (**b**) Δ = 2 mm, (**c**) Δ = 3 mm, (**d**) Δ = 4 mm, (**e**) Δ = 5 mm, (**f**) Δ = 6 mm. *h*_ef_ = 50 mm, *β* = 20°.

The sensitivity of the model to the finite element mesh discretization method was evaluated using 6 mesh types. The size of the mesh elements Δ was varied along the form of a hypothetical cone angle (inclined at an angle in the model, Figure 3). Changes in the characteristic dimension Δ of the elements were made in 1 mm increments, ranging from 1 to 6 mm. The simulation results are illustrated in Figure 6.

The analysis showed a strong correlation between the finite element mesh density in the model and the computation time, solution convergence and the quality of the failure trajectory representation. The model in Figure 6a requires significantly long computation time, but makes it easier to obtain a “smooth” crack surface. In the case of models in Figure 6c–f, the increase in the dimension Δ of the mesh elements causes a decrease in the accuracy of the gap representation. At the apex of the crack, the algorithm “stalls” and has a growing difficulty in correctly determining the potential crack direction of the material, leading to a halt in propagation. After evaluating the obtained solutions, it was decided to continue using the mesh according to model 6b in the analysis, i.e., with the characteristic dimension of the element on the side of the potential failure cone equal to Δ = 2 mm. 

The analysis above also shows that in the initial stage of crack growth in rock induced by the action of the undercutting anchor, there is mainly material tearing (Mode I). In the further stage of crack development, there is an additional effect of bending of the welded element (similar to bending of a unilaterally restrained beam). As a result, there is additionally a wall of material along the resulting crack (Mode II). This may explain the instability of calculations and the problems of the algorithm with determining the direction of failure propagation at the crack top. 

Finally, the parameters of anchor, rock in the rock-anchor contact were used for further analysis as follows:

Finite elements were used:

Sandstone: Element type: CAX4R: A 4-node bilinear axisymmetric quadrilateral, reduced integration, hourglass control. The finite element mesh was densified in the region of the predicted potential rock fracture (Based on existing fracture penetration models [35,36]).

Anchor: Element type: CAX4R.

Assumptions for simulation:

Type of material:

Sandstone: elastic, isotropic, quasi-brittle materials. Elastic Modulus–E = 14,276 MPa, Poisson’s ratio–ν = 0.247, Tensile Strength–σ_t_ = 7.4 MPa, 

Steel–material: elastic, isotropic, Elastic Modulus–E = 210,000 MPa, Poisson’s Ratio–ν = 0.3.

Damage initiation in rock material: maximal principal stress,

Damage evolution: type: energy, softening: linear. Damage for traction separation laws: maximal principal stress damage, fracture energy = 0.335 N/mm.

The interaction of the anchor with the rock was treated as a contact issue, with Coulomb friction. Interaction between rock and anchor: interaction type: surface to surface contact (standard), discretization method: surface to surface. Finite sliding. 

Applied friction coefficient between rock and anchor material (steel)–μ = 0.2. 

The simulation used an elastic, quasi-brittle material based on studies of the physical parameters of the rock in which the anchors were pulled. In compression tests, the rock material (sandstone) was found to crack rapidly (in an almost “explosive” manner) often leading to rapid specimen failure. In contrast, single (discrete) fractures were observed in the anchor pull-out tests. Thus, the failure mechanism corresponded to the behavior of elastic, quasi-brittle materials. The coefficient of friction in the rock anchor contact was taken as an average value from the literature data. 

**Figure 7:**
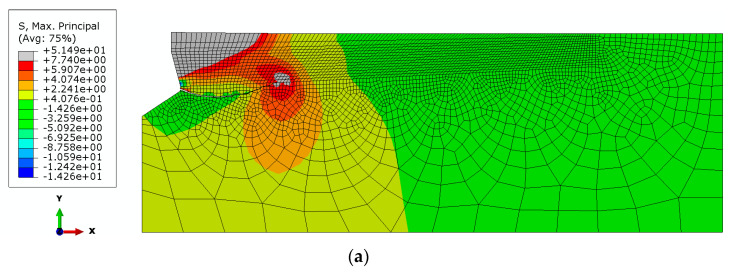

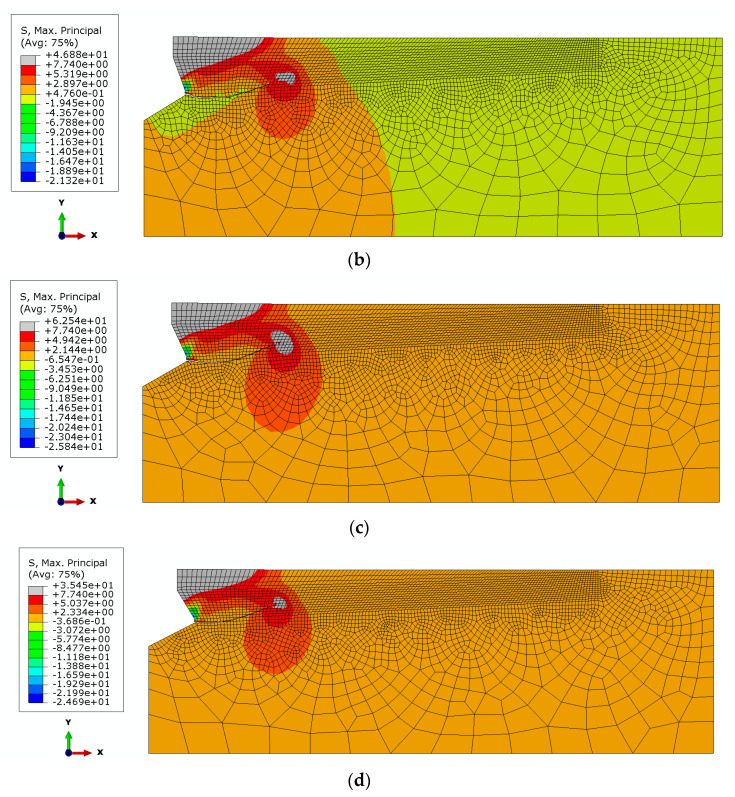
Distribution of maximum stresses and initial trajectory of sandstone cracking under the action of the undercut anchor head, for *h*_ef_ = 50 mm, *μ* = 0.2 and head angle: (**a**) *β* = 15° (**b**) *β* = 20°, (c) *β* = 25°, (**d**) *β* = 30°.

The distributions of the maximum principal stresses and the trajectories of the propagating fractures in the initial phase of the destruction of the rock medium continuity, for the considered cases of the undercutting head angle, are illustrated in Figure 7.

**Figure 8:**
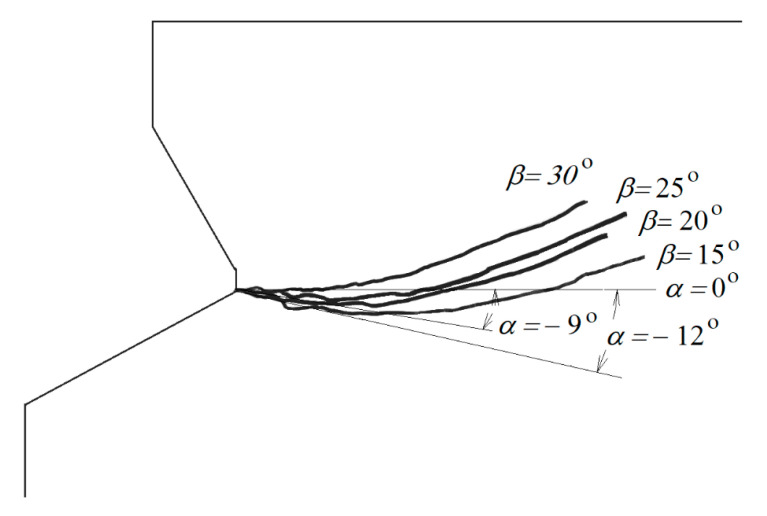
Trajectories of failure surfaces for considered angles of anchor head *β* (comparing with Figure 6) and example values of initial angle of failure propagation: *α* = −12° for *β* = 15° and *α* = −9° for *β* = 20°.

It can be seen from Figure 7 that an increasing angle of the undercutting head results in a lower-depth penetration of the crack. For comparison, Figure 8 collates the obtained material fracture trajectories for each case of undercutting head *β*. 

For angles *β* ≤ 25° the values of the initial angle of penetration of the failure surface of *α* ≤ 0° were obtained, which indicates a strong penetration of the deep failure zone of the medium and significant deformation of the medium in the plane of the axial section of the anchor. Potentially, the range of the failure zone of the medium together with the volume of detached lumps of material increase here. Due to the characteristics of the adopted model of the axisymmetric impact of the anchor on the rock material, it is difficult to draw conclusions about the behavior of the medium in the plane perpendicular to the anchor axis. Then, significant circumferential stresses are reported to occur, leading to cracks in the material in the plane of the model currently used. In order to clarify this issue, 3D FEM analyses and the use of non-linear material models are envisaged in future studies. 

## 4. Experimental Verification

In previously published studies [31,33], the radius of the support arrangement (R) of the anchor pull-out device (with three adjustable supports) was altered in the range of 300–390 mm (as required). The depth *h*_ef_ was ~50–140 mm. The value of the ratio of the radius of the supports to the depth of embedment was sufficiently large to avoid any potential influence of the supports on the free breakout capability of the rock medium. This was, therefore, a free surface breakout, and the term “free breakout failure” may be used. 

A number of tests were carried out on undercut anchors fixed in rock material obtained from sandstone seams or fixed directly in the natural rock mass. The arrangement of anchors fixed in the drilled holes was as close to parallel as possible to the predicted layering. Perpendicular stratification resulted in the extraction of a considerable size of rock material destruction.

A characteristic feature of the study was the high variability with respect to the condition and structure of the rocks or their compressive strength, depending on the particular mining site where the study was conducted.

In the laboratories of the Department of Geomechanics and Underground Construction of the Faculty of Mining and Geology of the Silesian University of Technology, the specimens were subjected to strength tests. The tests were in full compliance with the recommendations of the International Society of Rock Mechanics (ISRM) in terms of the accuracy of sampling, instruments and testing methodology. Complementary studies were conducted in the Department of Structural Mechanics; Faculty of Civil Engineering; Lublin University of Technology [38].

In the compression tests, measurement consistency *c* and internal friction angle *φ* were determined for the studied rocks. 

As a result of the tests carried out within the project, 115 successful solid rock breakout trials were made using a fixed undercut anchor. Results for the Braciszów mine are summarized in Table 1. The Braciszów mine contained dense, compact sandstone.

The mechanical parameters of the rock used in the FEM simulation are within the range obtained in the strength tests summarized in Table 1.

**Figure 9:**
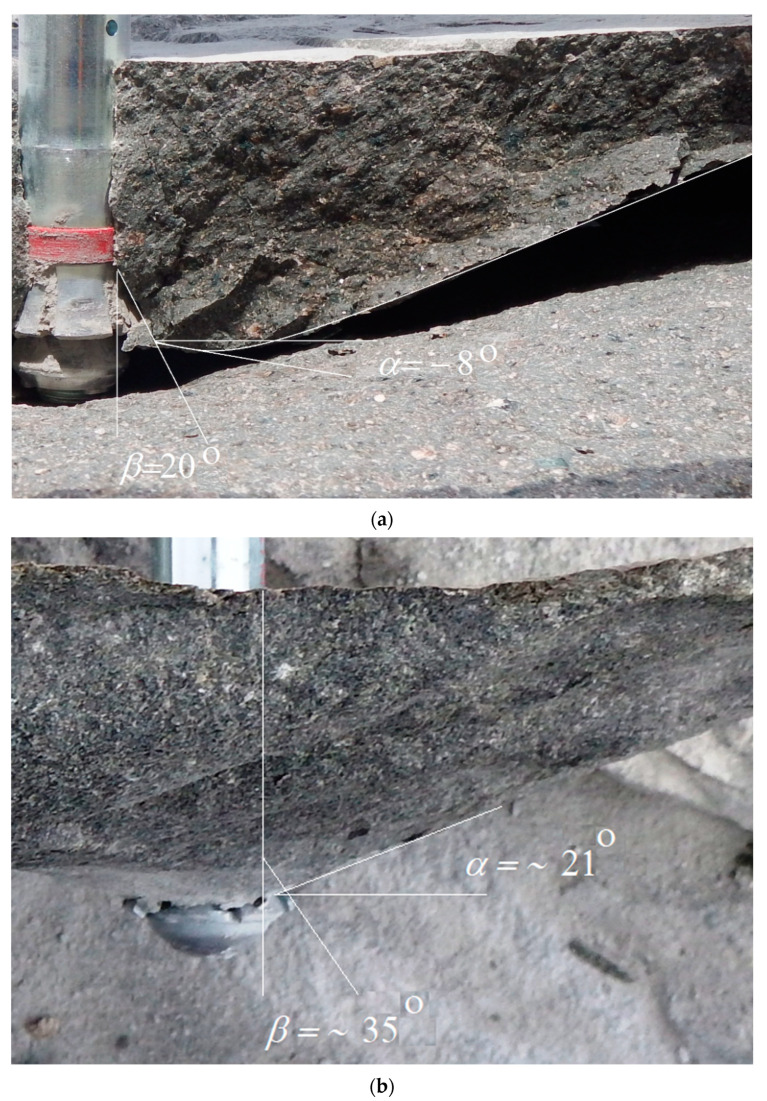
Initial crack penetration angle: (**a**) anchor M20, *α* = ~−8° for head angle *β* = ~20°; (**b**) anchor M12, *α* = ~21° for head angle *β* = ~35°.

As it can be seen from Figure 9, for M20 anchors it was possible to confirm the results of the FEM analysis. For the head angle *β* of about 20 the detachments are shown to propagate with an initial angle of failure penetration *α* of about ~−8°.

Figure 9a shows a simplified method for determining the angle of failure (detachment) at the initial stage of crack propagation. The envelope with an approximation of irregularities on the failure surface was made. The angle in question is between the tangent to the trajectory and the tangent to the anchor outline. 

There is a clear trend in the effect of head angle on the extent of the rock failure zone. The initial crack penetration angle is shown to depend strongly on the angle of the anchor’s undercutting head. Smaller angles (Figure 9a) are favorable for crack penetration in the initial phase of propagation and angles *α* with values below 0°. Larger head angles (Figure 9b) typically result in faster crack propagation towards the free rock surface and for destruction cone angles *α* larger than 0°. This is ultimately reflected in the extent of the destruction zone on the free rock surface. Therefore, as the field tests showed, smaller anchor head angles generally lead to larger rupture distances, while larger anchor head angles limit the extent of the failure zone.

In the analytical works only the initial stage of propagation is considered due to the fact that in FEM simulation, at a certain moment of simulation the propagation becomes unstable, at a point it stops or starts “keying” instead of penetrating towards free rock surface. ABAQUS program is not able to correctly determine the direction of propagation in the crack apex at this moment for an assumed material model. Hence, there was a need to stop the simulation and consider the angle of failure only at the initial stage. Observations made during field tests showed that the angle value in the initial phase of failure has a bearing on the final extent of the failure zone measured on the free rock surface. Negative angle values extend the damage zone further than in the case of angles greater than zero. 

The course of the damage trajectory presented in Figure 9, despite the large scattering of the field test results due to the heterogeneity of the rock structure, confirms the results of the numerical tests. However, comparable conditions of simulation and field tests must be maintained (homogeneity of rock structure, without internal cracks, gapping, etc.), or the correctness of crane installation. 

Under the conditions of field tests [31], the destruction surface mapped in the rock after the destruction cone had peeled off, was scanned, digitally processed, and smoothed using appropriate software. In the final stage, the crack trajectory was determined in the respective cross-sections, showing that the dimensions of the failure cone and the angle value of the failure cone depend, inter alia, on the embedment depth *h*ef. 

## 5. Discussion

Rock failure under the action of an undercutting anchor is a highly complex problem. Unlike in concrete, disturbances in the internal structure of rocks cause a significant scattering of results obtained during the determination of physical and mechanical parameters of the base material and in anchor pull-out tests [31,33,39]. Therefore, the main premise of the research conducted by members of the research team was to determine the tendency of changes in the process of rock destruction, which can be observed during changing physical and mechanical parameters of rocks, parameters of execution of the anchor pull-out process and changes of design parameters of the undercutting head. The paper [37] analyzed the influence of rock physical-mechanical parameters (Young’s modulus, Poisson’s number, tearing strength) and friction coefficient in the rock-anchor head contact. This paper presents the results of the analysis of the effect of the angle of the undercutting head on the course of the rock structure failure process. The analysis was conducted under the assumption that the length of the conical part of the undercutting head (L, Figure 3) is constant. This has a bearing on the magnitude of the projection of the conical surface in the plane perpendicular to the anchor axis, and on the development of stresses in the contact zone (this significantly affects the mechanism of load transfer from the anchor to the rock). Under field conditions, maintaining recommendations for the correct installation of the anchor in the hole is difficult and can significantly affect the actual value of the undercutting head angle. Therefore, this issue is addressed in this study.

Another study has been submitted for publication where, in turn, the effect of the wear process on the tapered part of the anchor is analyzed. This mainly leads to a decrease in *α* in the head angle. The length of the cone forming (*L*) also changes. As a result, there is a new issue of changing the contact conditions between the anchor and the rock, which was of interest. 

All analyses so far show a strong simultaneous influence of the above-mentioned influencing factors on the final effect, i.e., the value of the anchor pull-out force, the course and extent of the rock failure zone and the potential volume of rock fragments to be peeled off. The problem is very complex and requires extensive numerical and field studies.

Under field conditions, maintaining recommendations for the correct installation of the anchor in the hole is difficult and can significantly affect the actual value of the undercutting head angle. 

## 6. Conclusions

Under field test conditions, where it is difficult to maintain the imposed accuracy of the installation dimensions of the undercutting anchor head elements, different values of the head angle *β* can be expected. As a result, the course and extent of the failure zone can vary considerably, ultimately resulting in various volumes of rock breakout. Ultimately, this can also significantly affect the performance indicators of the proposed stripping process using undercut anchors. 

The study shows that the initial crack penetration angle significantly depends on the angle of the anchor undercutting head. Smaller angles tend to favor crack penetration in the initial phase of propagation and angles *α* with values below 0°. Larger head angles promote faster crack propagation towards free rock surface and for destruction cone angles *α* larger than 0°. This is ultimately reflected in the extent of the destruction zone on the free rock surface. Therefore, as the field tests showed, smaller anchor head angles generally lead to larger rupture distances, while larger anchor head angles limit the extent of the failure zone.

The presented research results provide new knowledge in the area of rock medium failure zone formation under the action of undercutting anchors. So far, the knowledge has mainly been concerned with the formation of failure of concrete, which is not adequate for the course of destruction of rock media. In terms of evaluating the extent of the failure zone, which is important for the unique stripping method considered in this paper, the previous treatment of this issue was an oversimplification (CCD method). This was demonstrated by detailed numerical analyses as well as field investigations by the authors of the study. The present study complements and extends previous analyses conducted by the team of authors of the study, especially [31,33,35,36,37,39].

## Figures and Tables

**Table 1:** The mechanical characteristics of rocks in Braciszów mines.

*f*_c_ (MPa)	Standard Deviation	*f*_t_ (MPa)	Standard Deviation	k = f_c_/f_t_	*φ* (°)	*c* (MPa)	*E* (GPa)	Standard Deviation	ν (-)	Standard Deviation	*E*_f_ (N/mm)	Standard Deviation
155.3	29.17	7.614	0.64	19.41	49.5	14.5	15.745	4.757	0.203	0.068	0.329	0.049

*f*_c_—compressive strength; *f*_t_—tensile strength; *c*—cohesion; *φ*—angle of internal friction; k—strength asymmetry factor; *E*_f_—fracture energy; *ν*—Poisson’s Ratio.

## Data Availability

The data presented in this study are available on request from the corresponding author.
